# Technology-Enabled Health Care Collaboration in Pediatric Chronic Illness: Pre-Post Interventional Study for Feasibility, Acceptability, and Clinical Impact of an Electronic Health Record–Linked Platform for Patient-Clinician Partnership

**DOI:** 10.2196/11968

**Published:** 2020-11-26

**Authors:** Lisa Opipari-Arrigan, Dana M H Dykes, Shehzad A Saeed, Sunny Thakkar, Lisa Burns, Barbara A Chini, Gary L McPhail, Ian Eslick, Peter A Margolis, Heather C Kaplan

**Affiliations:** 1 Anderson Center for Health Systems Excellence Cincinnati Children's Hospital Medical Center Cincinnati, OH United States; 2 Behavioral Medicine and Clinical Psychology Cincinnati Children's Hospital Medical Center Cincinnati, OH United States; 3 Department of Pediatrics University of Cincinnati College of Medicine Cincinnati, OH United States; 4 GI Care for Kids Atlanta, GA United States; 5 Gastroenterology and Nutrition Dayton Children’s Hospital Dayton, OH United States; 6 Gastroenterology and Nutrition Wright State University Dayton, OH United States; 7 Pulmonary Medicine Cincinnati Children's Hospital Medical Center Cincinnati, OH United States; 8 Vital Labs, Inc San Francisco, CA United States; 9 Perinatal Institute Cincinnati Children's Hospital Medical Center Cincinatti, OH United States

**Keywords:** health services research, mHealth, eHealth, patient engagement, chronic illness, mobile phone

## Abstract

**Background:**

Mobile health (mHealth) technology has the potential to support the Chronic Care Model’s vision of closed feedback loops and patient-clinician partnerships.

**Objective:**

This study aims to evaluate the feasibility, acceptability, and short-term impact of an electronic health record–linked mHealth platform *(Orchestra)* supporting patient and clinician collaboration through real-time, bidirectional data sharing.

**Methods:**

We conducted a 6-month prospective, pre-post, proof-of-concept study of *Orchestra* among patients and parents in the Cincinnati Children’s Hospital inflammatory bowel disease (IBD) and cystic fibrosis (CF) clinics. Participants and clinicians used *Orchestra* during and between visits to complete and view patient-reported outcome (PRO) measures and previsit plans. Surveys completed at baseline and at 3- and 6-month follow-up visits plus data from the platform were used to assess outcomes including PRO completion rates, weekly platform use, disease self-efficacy, and impact on care. Analyses included descriptive statistics; pre-post comparisons; Pearson correlations; and, if applicable, effect sizes.

**Results:**

We enrolled 92 participants (CF: n=52 and IBD: n=40), and 73% (67/92) completed the study. Average PRO completion was 61%, and average weekly platform use was 80%. Participants reported improvement in self-efficacy from baseline to 6 months (7.90 to 8.44; *P*=.006). At 6 months, most participants reported that the platform was useful (36/40, 90%) and had a positive impact on their care, including improved visit quality (33/40, 83%), visit collaboration (35/40, 88%), and visit preparation (31/40, 78%). PRO completion was positively associated with multiple indicators of care impact at 3 and 6 months.

**Conclusions:**

Use of an mHealth tool to support closed feedback loops through real-time data sharing and patient-clinician collaboration is feasible and shows indications of acceptability and promise as a strategy for improving pediatric chronic illness management.

## Introduction

### Background

Optimal management of pediatric chronic illness requires a different type of health care system [1,2]. Current models of care organized around treating acute, episodic conditions no longer meet the needs of the growing number of children with chronic health problems [3,4]. Although, organizationally, care is focused around the clinic visit, more than 99% of patients’ lives are spent outside of this context. The Chronic Care Model positions *informed, activated patients* and *prepared, proactive clinicians* as foundational to improved health outcomes. However, it is difficult for most clinicians to be informed about what happens outside of the clinic. In turn, most patients have neither easy, understandable access to their own clinical data nor systematic ways to reliably measure and communicate changes in daily functioning. This is a missed opportunity for optimizing health care. Failing to connect what happens day-to-day with clinical decision-making leads to reactive instead of proactive care based on hazy memories, missing information, and subjective impressions of *how things have been going.*

Mobile health (mHealth) technology offers the opportunity to address these gaps by expanding care beyond the boundaries of time-limited, infrequent clinical encounters to support data sharing, communication, and collaborative decision-making between patients and clinicians [5-9]. This potential has encouraged considerable attention from the health care and technology industries; however, we have yet to see mHealth have a transformative effect on chronic care delivery systems or outcomes. Although the number of digital health apps, portals, and dashboards is rapidly increasing, evidence of their impact has been mixed and modest [10]. Some studies have demonstrated that mHealth supports improved engagement and adherence. For example, text messaging interventions have boosted attendance at pediatric HIV appointments [11] and have increased adherence in pediatric asthma [12], type 1 diabetes [13], and cardiac care [14]. Electronic health portals that track patients’ symptoms and provide decision support have decreased clinic visits and school absences in pediatric ulcerative colitis [15] and decreased flares and missed parental work in pediatric asthma [16]. Yet, there are countless other examples of mHealth technologies that fail to improve self-management behaviors or clinical outcomes [9,10,16-21]. In fact, a comprehensive review of mHealth technology for chronic disease management found that just over half impacted adherence behavior and only 39% impacted clinical outcomes [10]. In addition, studies show that about half of the individuals that download health apps stop using them, most soon after initial use [22,23].

Both technological design elements and clinical care integration likely contribute to an mHealth tool’s success or failure [24]. Tools that only enable tracking and display of user data may build awareness but are likely insufficient to engage individuals in health behavior change [24-26] and surmount the burden of data entry [22]. Rather, mHealth tools that support the Chronic Care Model’s vision of closed feedback loops and patient-clinician partnership, through making data relevant, understandable, and actionable and by facilitating collaboration, are more likely to be adopted and retained [26,27]. Beyond the technology itself, purposeful integration with clinical care, so that both patients and clinicians understand the *why* and *how* of incorporating mHealth tools, is paramount to achieving impact [10,26,28].

### Objectives

The purpose of this study is to test the feasibility, acceptability, and short-term impact of using an mHealth tool to facilitate the Chronic Care Model’s vision of closed feedback loops and patient-clinician partnerships. As there was no available commercial platform that reliably enabled the functionality needed to support the Chronic Care Model’s approach to continuous care, we developed an mHealth tool with patient and clinician end users to support pediatric chronic care during and between clinical encounters. The *Orchestra* platform [29] was designed to enable proactive and collaborative chronic care through (a) real-time sharing and visualization of clinical and patient-generated (ie, patient-reported outcome, PRO) data, (b) automated symptom surveillance with actionable alerts to signal changes in patient status based on patient-generated data, and (c) collaborative previsit planning. As a first step, this prospective, preliminary pre-post study tested *Orchestra* in 2 pediatric chronic diseases (cystic fibrosis [CF] and inflammatory bowel disease [IBD]). We hypothesized that parents, patients, and clinicians would find *Orchestra* feasible and acceptable and that we would see signals of clinically meaningful impact.

## Methods

### Setting and Patient Population

This study was conducted within the IBD and CF clinics at Cincinnati Children’s Hospital (CCH; Ohio, United States) and was approved by the CCH Institutional Review Board (#2014-0975). We conducted the work at CCH because of our existing relationships with clinics that had an interest and ability to partner with us in this proof-of-concept study. We used a convenience sample of patients and parents from the CCH CF and IBD clinics. The CF and IBD clinics were chosen to ensure that our study included conditions that varied in disease course, management, and time demands associated with daily care. Eligible participants were patients with IBD or CF aged between 14 and 21 years and/or a parent or legal guardian of patients aged between 0 and 21 years. If a patient was less than 14 years old, only their parent was enrolled in the study and used the *Orchestra* app. If a patient was older than 14 years, the family decided if only the patient, only the parent, or both would enroll and use the *Orchestra* app. Participants were English speaking or reading and had a smartphone (iOS or Android) with a mobile data plan and/or internet connectivity.

### Study Design and Protocol

This was a preliminary, prospective pre-post, proof-of-concept study. Recruitment began in May 2015 and continued for 1 year. Follow-up data collection was completed in fall 2016. Eligible participants were identified via clinic rosters and were contacted by research staff, who were not part of the clinical team, before or at a clinic visit. Interested participants watched a 3-min video [30] describing *Orchestra* while waiting for their visit and then discussed with their clinicians how they might use *Orchestra* as part of their care. Following the visit, participants met with staff to sign the informed consent form, download the *Orchestra* mobile app, and customize settings. Participants were enrolled for 6 months.

### Orchestra Technology Platform

**Figure 1:**
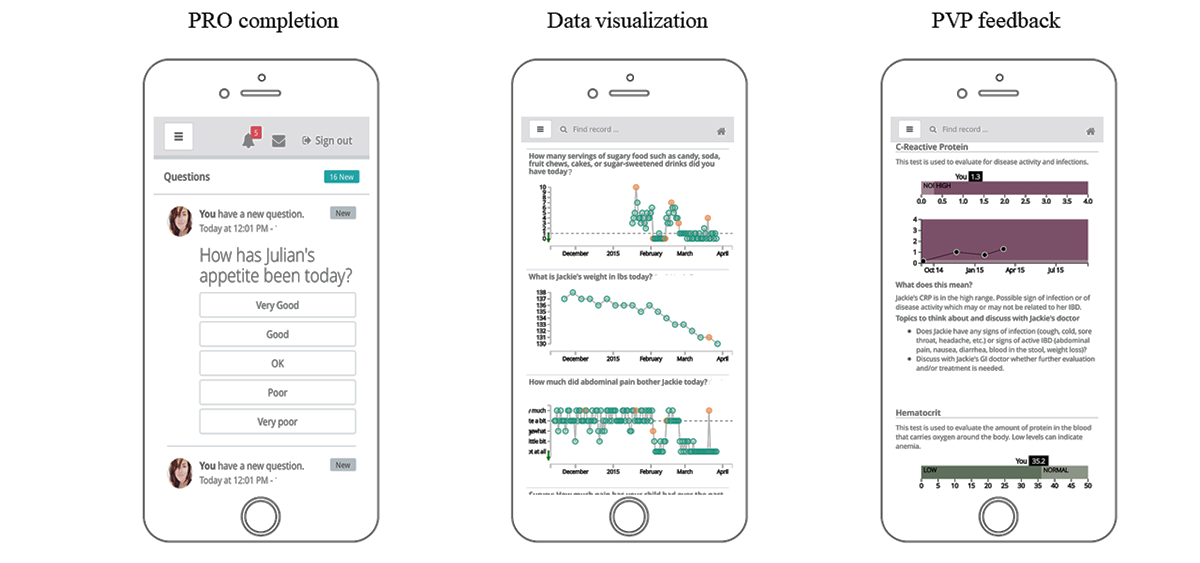
“Orchestra” participant mobile app example screens. PRO: patient-reported outcome; PVP: previsit plan.

The *Orchestra* technology platform [29] was developed with clinicians, patients, and parents via a user-centered, agile design process. The platform included a patient- or parent-facing mobile app and a linked clinician-facing web-based dashboard. Patient or parent functionality included the following (Figure 1):

- Library of PRO measures [31,32] that could be selected for symptom and general health tracking based on patient goals and customized for daily or weekly completion. There were 35 predetermined measures available for IBD (eg, abdominal pain frequency, stool consistency, and fatigue) and 28 available for CF (eg, energy level, cough severity, and appetite). Participants could also create custom symptoms with their clinician if needed (Multimedia Appendix 1).
- Real-time visualization of PRO data.
- PRO data point annotation and journal entry features for recording observations.
- Previsit health report showing disease-specific laboratory data (ie, C-reactive protein and hematocrit) and health metrics (ie, weight and pulmonary function tests) with simple visualizations and text descriptions of results and trends as well as personalized suggestions for topics to discuss with clinicians.
- Previsit plan (PVP) completion to inform clinical teams about patient symptoms, goals, and questions before a clinic visit.

**Figure 2:**
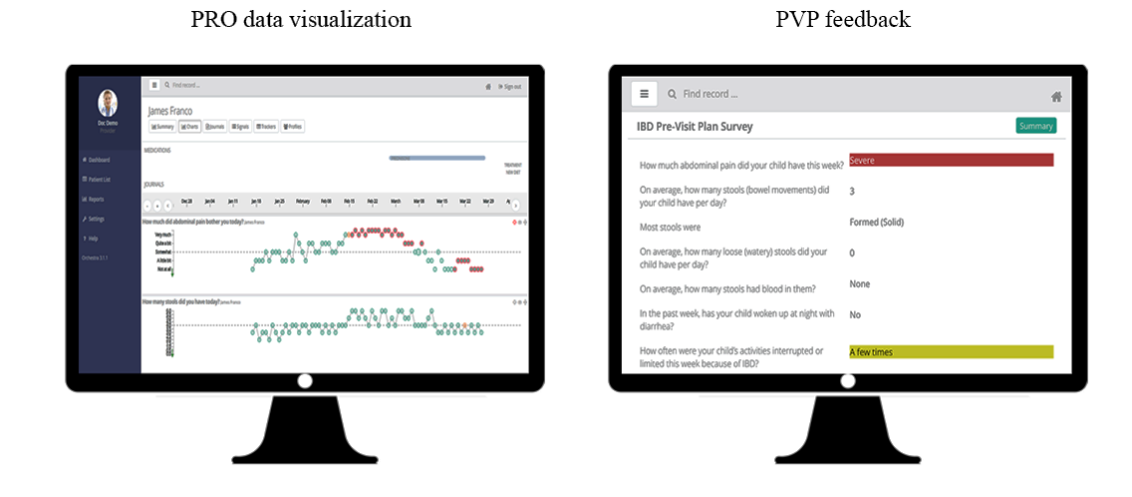
“Orchestra” clinician dashboard example screens. PRO: patient-reported outcome; PVP: previsit plan.

The clinician-facing web-based dashboard was accessed via Epic (Epic Systems Corporation), the electronic medical record, using a customized configuration that enabled one-click, single sign-on authentication. Clinical users included physicians and nurses in IBD and physicians, nurses, dietitians, a social worker, a respiratory therapist, and a psychologist in CF. The clinician dashboard enabled the following (Figure 2):

- Real-time visualization of PRO data and review of PVPs.
- Addition of notes to a patient’s account visible by the patient or parent and other clinical staff.
- Notification if a patient’s symptoms met prespecified criteria based on PRO data. Alert triggers were customizable and based on meeting a threshold value and/or meeting standard statistical process control criteria of 8 data points above or below the median after a 20-data point baseline was established [33]. If alerts were triggered, clinicians were notified via the *Orchestra* dashboard and email.

### Orchestra Intervention

Use of the *Orchestra* technology platform was accompanied by a delivery system intervention to integrate the technology into clinical workflows and patients’ lives, personalize goals for use, and establish accountability for system use between participants and clinicians. All clinicians who cared for patients, including physicians, nurses, dietitians, a respiratory therapist, a social worker, and a psychologist, received training on the use of *Orchestra* [29]. Participants and clinicians used shared decision-making during the baseline clinic visit to discuss their preferences and goals for *Orchestra* use, select which PROs to complete and at what frequency, and decide who (patient and/or parent) would use the tool. Clinicians discussed health concerns and goals with patients (eg, reducing symptoms, reducing medications, and preventing exacerbations) and identified whether using *Orchestra* would help achieve these goals. The clinician and family then reviewed the PRO and symptom measures available in *Orchestra* and determined which measures were most relevant to addressing the identified goals. They also established a *social contract* to set expectations regarding between-visit data completion (by participant), review (by clinician), and communication (participant-clinician). Clinicians chose whether to set data alerts and customized trigger points based on the PROs selected and patient needs.

To reinforce the use of *Orchestra* by demonstrating that data collected between visits were reviewed, a designated clinical team member was asked to review the data entered into the *Orchestra* platform each week and within 1 day of receiving a data alert. In addition, clinicians were asked to review PVP and PRO data before all clinic visits. At visits, clinicians were encouraged, at least, to verbally acknowledge reviewing the data and ideally to use the data for discussion and decision making.

### Data Collection

Participants completed study assessments at baseline and 3- and 6-month follow-up visits during regularly scheduled clinic appointments. As this tool was designed to be used as part of clinical care, if a patient did not have a clinic appointment within ±1 month of a follow-up time point, the assessment was not completed. Participants were still included in the study if they did not have an appropriately timed follow-up visit, but that specific assessment was considered missing.

Health status indicators, including the Physician Global Assessment (PGA) Score (IBD) and forced expiratory volume in 1 second (FEV1) percent predicted (CF), were collected from the electronic health record to understand the baseline health of participants. The PGA score is an indicator of disease severity that is rated at each clinic visit by the physician and is based on symptoms, clinical exam, and laboratory measures [34]. PGA is used to classify disease as in remission, mild, moderate, or severe. FEV1 is calculated as part of pulmonary function testing and is an established marker of disease progression in CF [35]. The lower the FEV1 percent predicted, the more severe the lung disease.

Clinicians and research staff reported on baseline technology discussion and set-up time via a paper and pencil measure completed immediately following the patient visit. In addition, the patient’s physician and, if applicable, a second clinical team member involved in using Orchestra completed surveys after each visit regarding their assessment of the patient and parent’s engagement in the visit and their experience using Orchestra. However, due to an inability to obtain responses in a timely manner from clinicians, the data were not analyzed.

### Outcomes

The *feasibility* of implementing and supporting *Orchestra* in care was assessed by measuring the percentage of eligible patients with access to a smartphone with data plan or internet connectivity, minutes spent discussing *Orchestra* (self-reported by the clinician at baseline), minutes spent enrolling and setting up the *Orchestra* app (self-reported by research staff), and participants’ report of whether *Orchestra* was used during follow-up visits. One facet of *acceptability* was assessed by measuring usage of the app including PRO completion (ie, percentage of PROs a participant answered), weekly engagement with the mobile app (ie, percentage of weeks a participant input data at least once), and PVP completion (percentage of PVPs that were submitted in the app). We examined the short-term *impact* of *Orchestra* on disease self-efficacy, visit engagement, and care impact, including perceived care quality, patient-clinician collaboration, visit preparation, disease insight, treatment plan quality, and perceived usefulness. As we did not expect health outcomes to change over the 6-month study period, we focused on measures of short-term meaningful clinical impact that reflected participants’ perceptions of their ability to manage their illness (efficacy) as well as their perceptions of their clinic visit and interaction with their clinicians. Disease self-efficacy was measured using the validated Self-Efficacy for Managing Chronic Disease 6-Item Questionnaire [36,37]. Each item uses a 1 (*not at all confident*) to 10 (*totally confident*) scale, and a mean of all items is calculated. Visit engagement was measured using an 8-item survey examining visit preparation, knowledge, and involvement given at baseline and at the 3- and 6-month follow-up (Multimedia Appendix 2). As one item assessed the use of tracked data at clinic visits, it was assessed only at 3 and 6 months and was not included in the analysis. At 3 and 6 months only, participants completed a 20-item care impact survey that measured the impact of *Orchestra* on care quality (4 items), patient-clinician collaboration (2 items), visit preparation (6 items), disease insight (3 items), treatment plan quality (3 items), and perceived usefulness (2 items; Multimedia Appendix 2). Both the visit engagement and care impact surveys were developed theoretically by a team of experts for this study, used a 6-point scale ranging from 1 (*strongly disagree)* to 6 (*strongly agree)*, and were tested in a convenience sample for understandability. The 3- and 6-month surveys also included a single question measuring overall satisfaction: “How likely are you to recommend Orchestra to other parents/patients like you?”, which respondents answered on a 10-point scale from 10 (*very likely*) to 1 (*not at all likely*). Impact on the treatment plan was measured by chart review examining the percentage of data alerts that led to documented clinical action including phone calls, emails, or messages to check status; expedited clinic appointments or laboratory tests; referrals; or treatment changes.

### Statistical Analyses

Descriptive statistics, including means and ranges for continuous variables and percentages for categorical variables, were used to describe the study population, feasibility, acceptability, satisfaction, and perceived impact. A mean score of 4 or higher on the visit engagement survey and care impact survey subscales was defined as an indicator of engagement in the visit and improvement in care experience, respectively. *t* tests were used to assess the impact of condition (CF or IBD), person using *Orchestra* (parent or patient), and baseline health status in CF (FEV1: ≥70% predicted or <70% predicted) on PRO completion. Cohen *d* measured the effect size. Univariate analysis of variance tests was used to evaluate differences in acceptability measures across study clinicians and across IBD baseline health status (PGA: continuous remission, remission, mild, and moderate). Paired *t* tests examined the impact on disease self-efficacy and visit engagement from baseline to 6-month follow-up. Pearson correlation coefficients examined the relationships between PRO completion and perceived impact on care. Analyses were conducted using the Statistical Package for the Social Sciences v23 (SPSS, IBM Corp).

We aimed to enroll 100 participants. Our target was based on knowledge of the eligible population in each clinic, frequency of clinic visits, available research resources, and a desire to complete the study while the technology remained current. As efficacy was not the primary endpoint, we did not perform an a priori power calculation.

## Results

### Overview

**Figure 3:**
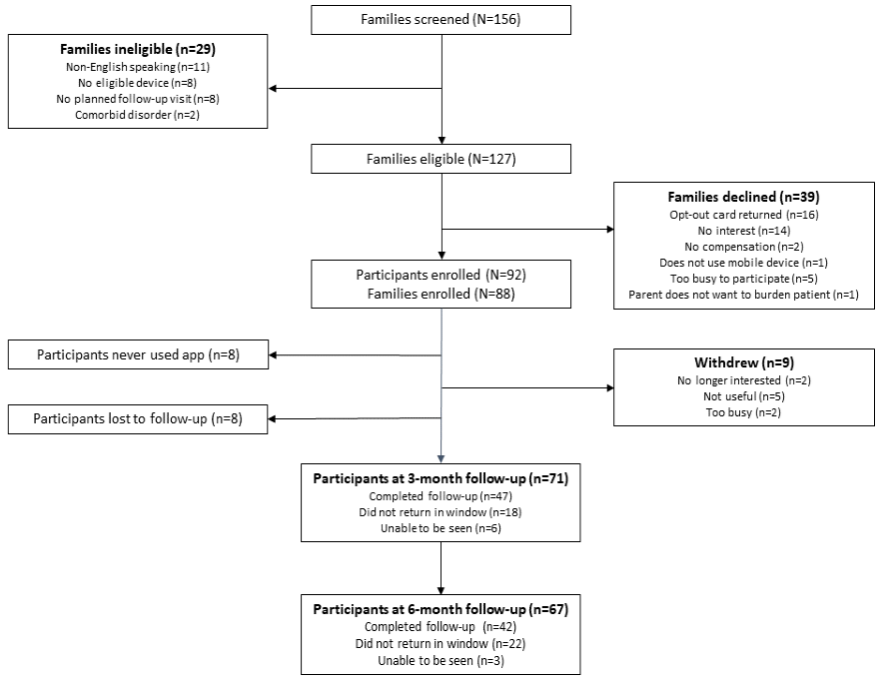
Participant flow diagram. Of the 127 eligible families, 88 were enrolled (92 participants). At 3 months, 77% (71/92) of participants remained in the study and 66% (47/71) completed the 3-month follow-up within the specified study time window. At 6 months, 74% (67/92) of the participants remained in the study and 63% (42/67) completed the 6-month follow-up within the specified study time window.

Of the 127 families approached, 88 (69.2%) agreed to participate. A total of 92 participants (CF: n=52 and IBD: n=40) from 88 families enrolled in the study (Figure 3). In 38 families (38/88, 43%), an adolescent patient participated; in 46 families (46/88, 52%), a parent participated; and in 4 families (4/88; 5%), both participated. At 3 months, 71 of the original 92 participants (77%) remained in the study; at 6 months, 67 of the original 92 participants (73%) remained (Figure 3). The 6-month retention rate was higher among IBD clinic participants (32/40, 80%) than among CF clinic participants (35/52, 67%). Eight different attending physicians had participants enrolled on the platform, 2 from IBD and 6 from CF. This included all attending physicians in the CF clinic and the physicians who most frequently saw patients with IBD in the gastroenterology clinic. Table 1 describes the demographic and health status characteristics of the participants. Characteristics of the participants mirrored the general demographics of the clinic populations. Participants selected an average of 4.96 PROs (range: 1-13) to track in the app.

**Table 1:** Baseline demographic and health status characteristics of the patients for each enrolled family.

Patient characteristics			Cystic fibrosis (n=51)		Inflammatory bowel disease (n=37)		Full sample (N=88)
**Patient age (years), mean (SD)**			10.55 (6.01)		15.70 (4.02)		12.72 (5.82)
	0-2, n (%)	7 (14)		1 (3)		8 (9)	
	3-12, n (%)	24 (47)		9 (24)		33 (38)	
	12-21, n (%)	20 (39)		27 (73)		47 (53)	
**Patient gender, n (%)**							
	Female	24 (47)		22 (60)		46 (52)	
	Male	27 (53)		15 (40)		42 (48)	
**Patient race, n (%)**							
	White	49 (96)		35 (95)		84 (96)	
	Black or African American	0 (0)		2 (5)		2 (2)	
	Other	2 (4)		0 (0)		2 (2)	
**Disease indicators, mean (SD)**							
	Mean forced expiratory volume in 1 second percent predicted^a,b^ (cystic fibrosis only, n=38)	96.89 (19.69)		—^c^		—^c^	
	Disease type (inflammatory bowel disease only)	—^d^		Crohn: 29 (78%) Ulcerative Colitis: 8 (22%)		—^d^	
	Physicians global assessment score (inflammatory bowel disease only)	—^d^		Continuous Remission: 10 (27%) Remission: 14 (38%) Mild: 11 (30%) Moderate: 2 (5%)		—^d^	
**Family income (US $), n (%)**							
	<25,000	4 (8)		3 (8)		7 (8)	
	25,000 to 49,999	12 (24)		1 (2)		13 (15)	
	50,000 to 74,999	8 (15)		5 (14)		13 (15)	
	75,000 to 99,999	9 (18)		7 (19)		16 (18)	
	≥100,000	15 (29)		14 (38)		29 (33)	
	Unknown	3 (6)		7 (19)		10 (11)	

^a^Scores ranged between 48 and 127.

^b^Not all participants were old enough to reliably complete pulmonary function testing.

^c^Measure not applicable for patients with inflammatory bowel disease.

^d^Measure not applicable for patients with cystic fibrosis.

### Feasibility

Access to technology was not a significant barrier to participation. Of the families approached, 119 of 127 (94%) had regular access to an eligible device and internet connectivity. Most participant-clinician conversations about *Orchestra* (40/83, 48%) required 5-10 min, with 34% (28/83) taking <5 min and 18% (15/83) requiring >10 min. *Orchestra* enrollment and app installation took 14 min on average (SD 7; 95% CI 13-16), ranging between 2 and 43 min (data available for n=84 families). Longer set-up times were related to technical issues or need for more thorough instruction. It was feasible to incorporate *Orchestra* into follow-up clinic visits—participants reported discussing *Orchestr*a in 83% (34/41) of visits at 3 months and in 71% (29/41) of visits at 6 months.

### Acceptability

Among those who used the PRO feature (n=80), 19,954 PROs were completed. The average PRO completion rate by participants was 61% (range: 6%-100%; 95% CI 55-68). There was no difference in mean PRO completion rates between CF (n=47, 58%; 95% CI 50-67) and IBD (n=33, 66%; 95% CI 58-75; *P*=.19). Parents (n=41) had significantly higher rates of PRO completion (67%; 95% CI 59-76) than patients (n=39; 55%; 95% CI 46-64; *P*=04; Cohen *d*=0.46). Participant PRO completion rates varied significantly across different physicians (n=8), ranging between 38% and 77% (*P*=.02; partial eta squared=0.20). PRO completion was not related to baseline health status in CF (FEV1 percent predicted) or IBD (PGA score), data not shown.

Average weekly engagement, a measure of app *stickiness* that indicates the participants’ willingness to return to the app, was 80% (n=80; range: 21%-100%; 95% CI 75-85). No significant differences in weekly engagement were observed between CF (n=47; 80%; 95% CI 73-87) and IBD (n=33; 80%; 95% CI 72-88; *P*=.99) or based on participants’ physicians (n=8; *P*=.06). Parents (n=41) had a higher rate of weekly engagement (87%; 95% CI 81-92) compared with patients (n=39; 73%; 95% CI 65-81; *P*=.005; Cohen *d*=0.64). Patients with IBD in continuous remission for ≥1 year based on PGA (n=6) showed lower weekly engagement (55%; 95% CI 28-83) than patients in current remission for <1 year (n=13; 84%; 95% CI 71-96) or those rated as having mild (n=12; 86%; 95% CI 75-98]) or moderate (n=2; 93%; 95% CI 2-184) disease activity (*P*=.02; partial eta squared=0.28). No differences were found in the average weekly engagement based on FEV1 status in CF (n=35).

Participants received three PVPs on average (range: 0-6) and completed 49.4% (94/190). Most families who received a PVP (56/76, 74%) completed the information at least once.

### Short-Term Impact

Participants reported significant improvement in disease self-efficacy from baseline (7.90, SD 1.63; 95% CI 7.70-8.34) to the 6-month follow-up (n=40; 8.44, SD 1.34; 95% CI 8.01-8.87; *P*=.006; Cohen *d*=0.36). The majority of participants reported feeling engaged in their clinic visit at baseline (87/92, 95%) and 6-month follow-up (38/40, 95%), and there was no change in self-reported visit engagement scores from baseline (5.13, SD 0.74; 95% CI 4.84-5.22) to 6-month follow-up (n=40; 5.29, SD 0.66; 95% CI 5.06-5.50; *P*=.12). Most participants reported that incorporating *Orchestra* into care was useful and had a positive impact on care quality, visit collaboration, participant visit preparation, participant disease insight, and treatment plan quality. Table 2 shows the percentage of participants who responded that *Orchestra* improved their care experience. Participants who used *Orchestra* more were more likely to report greater perceived impact on care. As shown in Table 3, the higher a participant’s PRO completion, the more useful they found *Orchestra* and the more impact it had on reports of care quality, visit collaboration, participant visit preparation, and treatment plan quality at 3- and 6-month follow-up visits.

**Table 2:** Percentage of Orchestra users reporting that the tool improved their care experience.

Impact on care experience	3-Month follow-up (n=44)^a^, n (%)	6-Month follow-up (n=40)^b^, n (%)
Improved care quality	40 (91)	33 (83)
Improved collaboration	37 (84)	35 (88)
Improved participant preparation for visit	35 (80)	31 (78)
Improved participant disease insight	33 (75)	29 (73)
Improved treatment plan	38 (86)	32 (80)
Perceived useful for visit	40 (91)	36 (90)

^a^44 out of 47 participants completed the care impact survey at the 3-month follow-up.

^b^40 out of 42 participants completed the care impact survey at the 6-month follow-up.

**Table 3:** Pearson correlation coefficients between patient-reported outcome completion rate and impact of Orchestra on care experience.

Impact on care experience	3-Month follow-up (n=43)^a^	*P* value	6-Month follow-up (n=39)^b^	*P* value
Improved care quality	0.52	<.001	0.33	.04
Improved collaboration	0.39	.009	0.38	.02
Improved participant preparation for visit	0.47	.002	0.34	.04
Improved participant disease insight	0.33	.03	0.07	.67
Improved treatment plan	0.51	<.001	0.36	.03
Perceived useful for visit	0.51	<.001	0.22	.18

^a^44 out of 47 participants completed the care impact survey at the 3-month follow-up, and 43 out 47 participants received regular patient-reported outcomes (n=1, used tool for previsit plan only).

^b^40 out of 42 participants completed the care impact survey at the 6-month follow-up, and 39 out of 42 participants received regular patient-reported outcomes (n=1, used tool for previsit plan only).

Participants reported moderately high satisfaction (ie, likelihood of recommending to others) with using *Orchestra* at the 3-month (mean 8.16, SD 1.76; n=45) and 6-month follow-ups (mean 8.55, SD 2.05; n=40).

Clinicians set optional alerts for 49 of 92 (53%) participants. A total of 192 alerts were triggered among 35 of the 49 (71%) patients. On the basis of the clinician report and medical record review, 39% (74/192) of alerts led to documented, actionable clinician behavior. For example, in one case, the CF team was signaled about a change in mucous production and cough based on *Orchestra* alerts. This prompted a phone call to the family who had noted the symptoms but had not acted on them. The clinical team was able to initiate early treatment (antibiotics and increased airway clearance) and potentially avoid an inpatient admission for pulmonary exacerbation. More than half (65/111, 59%) of alerts in IBD led to action as compared with only 11% (9/81) in CF.

## Discussion

### Principal Findings

We tested a technology platform and care delivery system intervention designed to support the Chronic Care Model’s vision of informed patients and prepared clinicians working in partnership with closed feedback loops. This proof-of-concept study suggests that the mHealth platform is feasible, acceptable, and shows promise for clinically meaningful impact. The average PRO completion rate by participants across the 6 months of the study was 61%, and the average weekly platform use was 80%, suggesting reasonable acceptability. Participants reported significant improvement in self-efficacy from baseline to 6 months. At 6 months, most participants reported that the platform was useful and had a positive impact on their care, including improved visit quality, visit collaboration, and visit preparation. PRO completion rate was moderately associated with participant-reported visit preparation, care quality, treatment plan quality, collaboration, disease insight, and usefulness at 3 months. All relationships, except disease insight and usefulness, remained significant, although weaker, at 6 months.

The addition of shared mHealth technology into the process of managing chronic disease enabled frequent, timely, and problem-focused information and communication. We posit that these types of real-time, bidirectional shared data potentiate a fundamental shift in the interaction between clinician and patient—a shift from clinician as the expert delivering care to a relatively passive patient, to clinician and patient contributing to shared work in which the expertise of both is needed. Our findings are consistent with the small number of mHealth studies specifically designed to facilitate patient-clinician collaboration that also show early evidence of effectiveness [16,38] and, as hypothesized, demonstrate that collaboration is associated with sustained use [26]. For example, a pilot study compared pediatric patients with asthma receiving standard care versus access to a portal that included symptom tracking, sharing of concerns and goals, and symptom threshold–based prompts [16]. In this study, more than half of the parents completed >80% of monthly surveys, and they reported improved communication, fewer asthma flares, and less missed work compared with standard care. *Orchestra* achieved similar rates of engagement (80% average weekly engagement) and sustained use (almost 60% of participants engaged for ≥24 weeks). Our work extends these results by demonstrating high rates of use in two other chronic pediatric diseases. We were also able to show that incorporating processes to support patient-clinician collaboration and closed feedback loops, including shared decision-making to establish goals for technology use and reinforcement of PRO completion through regular review and use of the data, is feasible to implement in a busy clinical setting. The benefits of focusing on mHealth users’ goals and their commitment to these goals were also underscored in a longitudinal study of mHealth app usage. This study showed that the key to successful use of mHealth technology revolved around persisting at goals while using the right system that fits users’ needs [23].

A key strength of our approach was that we focused not only on user-centered technology but also on the processes to support integration into workflow, closed feedback loops, and patient-clinician collaboration. This attention to helping our end users (ie, patients and clinicians) understand the value of between-visit data and feel comfortable with using it was critical to the tool’s acceptability and sustained use. Although mHealth apps alone often function as sophisticated data collection and delivery mechanisms, when well-integrated into care, they may support patients and clinicians in moving beyond traditional role expectations to learning to work together in new ways that transform their interactions, health care, and outcomes [39,40].

Given that participants were delivered PROs to complete once per day, the greater than 60% PRO completion rate seen in this study means that participants recorded health data on average about 4 days per week between clinic visits, resulting in a significant amount (almost 20,000 completed PROs) of previously untapped information that was available to inform care. Although technology enables easy data collection, more data are not always better, and it can easily overwhelm clinical teams [9]. Giving clinicians the ability to set data alerts was done as a way to help manage the volume of data in busy clinical workflows by highlighting potentially actionable information. Incorporating statistical process control methods to assist with identifying meaningful changes from the patient’s unique baseline was a novel approach. Although alerts were only set for about half of the patients, almost 40% of the alerts that fired led to documented action between clinic visits. Interestingly, more alerts were acted upon in IBD versus CF. Several factors may explain this finding, including more experience among our IBD physicians in selecting and using daily PROs; the complex, multisystem nature of CF; and the sensitivity of the alert threshold chosen by the physicians. Additional work is needed to understand how to balance sensitivity to detect meaningful changes in patient status with the volume of alerts generated. We speculate that the right balance will differ by condition and by patient.

Our data suggested that participants who used *Orchestra* more reported greater perceived impact on care. We found that the higher a participant’s PRO completion rate, the more they reported that *Orchestra* had a positive impact on care quality, visit collaboration, participant visit preparation, and treatment plan quality. We hypothesize that PROs, PVP data, real-time visualizations, and alerts better enabled patients and clinicians to work together between and during visits, leading to improvements in short-term outcomes. Specifically, we posit that by responding to daily or weekly PROs, participants had a systematic way to learn, remember, and communicate with clinicians between visits. In turn, the data potentiated clinicians being more informed during clinical contacts and better able to proactively address problems between visits. Another reason participants who completed more PROs may have reported greater benefits relates to the potential for closed feedback loops that reinforce platform use. Use of shared decision-making at the onset may have increased the likelihood that the data collected had relevance to both the clinician’s and participant’s goals, creating greater potential for relevant, actionable data. In turn, the potential for relevant, actionable data could have increased the probability that participants entered it and that clinicians reviewed it and talked about it at visits (reported in 83% of visits at 3 months and in 71% of visits at 6 months). As clinicians used tracked data, we believe that participants’ efforts were reinforced, leading to sustained data collection and more actionable results. A stronger relationship between tool use and care impact was found at 3 months as compared with 6 months. We hypothesize that this may be related to the length of the intervening time between the goal for tool use being discussed and PROs selected and the subsequent clinic visit. Patients and clinicians determined how and why the tool would be used at baseline only. It may be that 6 months later, the data are less valuable due to essential learning already occurring (perhaps at a prior visit) and/or areas of concern changing. More frequent recalibration between patients and clinicians (ie, every visit) may be needed to sustain the relevance of mHealth tool use in chronic care.

Our findings reveal several factors that may contribute to improved engagement with mHealth technology. Involvement of the clinical team in supporting participants’ platform use is critical [26]. Differences in PRO completion rates across physicians and a tendency for participants’ weekly engagement to vary by physician is consistent with observations that clinicians varied in skill at helping patients identify goals for tool use and integrating *Orchestra* data into follow-up visits. Understanding how to help clinicians maximize patients’ engagement and use of mHealth technology will be important for future interventions. Not surprisingly, parents showed higher levels of overall PRO completion and weekly app engagement as compared with teens. However, teens completed over half of the PROs they were sent. With realistic expectations for app use, this type of mHealth technology could support adolescent care transition. The finding that health status predicted weekly engagement in IBD but not CF suggests that disease course may lead to different patterns of app use. In IBD, participants in continuous remission used the app less than those who were not. Frequent PRO completion during periods of sustained wellness is likely a low-value behavior. In CF, a condition with periods of exacerbation but no remission, app use was not related to health status, suggesting that PRO completion behavior may be influenced by the interplay of disease course and current functioning. Although these two conditions differ significantly in required time for daily self-management, with CF typically being more intensive, average app use across the diseases did not differ, suggesting feasibility even in the context of high disease self-management demands.

### Limitations

This study had some limitations. Our evaluation of app use and impact is based on participants’ perspectives only. We attempted to collect clinician data on tool use and impact following clinical encounters but were unsuccessful in getting clinicians to complete surveys in a timely manner. Rather than asking physicians to complete the surveys immediately after the visit, we allowed them to answer them later by email. In future work, we will gather the data as soon as the physician leaves the clinic room or consider obtaining clinicians’ perspectives via qualitative interviews. As this study focused on proof of concept, we did not include a contemporaneous control group, so it is unclear how those receiving standard care felt about their visits and ability to communicate with their providers in between visits or whether self-efficacy would have gradually improved over time even without the use of *Orchestra*. It is not possible to rule out that either natural history led to the clinical impact observed or that selection bias led to those who were most interested in technology and collaboration choosing to participate, stay in the study, and show benefit. By using limited exclusion criteria, a pragmatic approach that utilized existing clinic visits (rather than research study visits), and studying more than one chronic disease, we attempted to lessen the impact on these potential threats to validity. Although our participants spanned the economic spectrum, one-third of the families included in this study had incomes above US $100,000. Although convenience sampling made sense in this early work, in future studies, using sampling techniques to ensure appropriate representation across the economic spectrum is critical. It will also be important to ensure that this type of technology and intervention does not increase health disparities. Some measures were developed for this study because of the lack of validated instruments to evaluate visit engagement and the impact of the mHealth tool on care. These measures had face validity and were tested for understandability, but we did not conduct psychometric testing to examine factor structure, reliability, or validity. As this was designed as a pragmatic study, we only attempted to collect follow-up data from participants if they had a clinical visit during the follow-up window. As a result, we do not have the follow-up questionnaire data on the full sample at either time point. For example, at 6 months, approximately two-third of the participants (42 of 67) who remained in the study had a clinic visit. There could have been systematic differences between participants who returned to the clinic more and less often. However, given that the intent of the study was as a proof of concept, identifying a tool that may have had a meaningful impact for even a fraction of the patients who used it is beneficial learning.

### Lessons Learned

The lessons learned in this process are important for future work on mHealth tools for chronic illness. First, the value of working iteratively and in concert with patient and clinician end users cannot be overstated. Through collaborative development, they showed us their pain points and thus the necessary functionality to sustain engagement. Second, the one-click, single sign-on with the electronic medical record was critical for our clinicians to sustain use. Although for a short time they were excited by the novelty of a new platform, its use was not sustainable in their workflow if it was not reachable from the electronic medical record. Third, the automated data signals were critical in facilitating clinician comfort with between-visit data collection and thus having multiple patients on the platform. Fourth, both clinicians and patients needed time to learn the value of between-visit data. Structuring workflow to encourage using the data facilitated the learning and was key. Finally, reliable and low-friction technology is necessary, but not sufficient, to support engagement over time. Rather, understanding patients’ and clinicians’ needs and goals and then using technology to support achieving them is essential for impacting health care and outcomes.

### Conclusions

We have shown that the use of an mHealth technology designed to facilitate the Chronic Care Model’s vision of closed feedback loops and patient-clinician partnerships is feasible, acceptable, and shows promise as a strategy for improving pediatric chronic illness management. These encouraging early results reinforce the potential of mHealth technology to support collaboration and real-time data sharing. Although the start-up company that developed *Orchestra* is no longer supporting the platform, this proof-of-concept study identifies key components necessary in any mHealth platform designed to support continuous data and patient-clinician collaboration, important processes necessary to include in clinician and patient workflows to facilitate technology use, and lessons for future studies.

## Appendix

Multimedia Appendix 1 Library of patient-reported outcome measures for cystic fibrosis and inflammatory bowel disease.

Multimedia Appendix 2 Parent visit engagement and parent care impact surveys.

## Abbreviations

CCH: Cincinnati Children’s Hospital
CF: cystic fibrosis
FEV1: forced expiratory volume in 1 second
IBD: inflammatory bowel disease
mHealth: mobile health
PGA: Physician Global Assessment
PRO: patient-reported outcome
PVP: previsit plan
