# Supplementary material for: Technology-Enabled Health Care Collaboration in Pediatric Chronic Illness: Pre-Post Interventional Study for Feasibility, Acceptability, and Clinical Impact of an Electronic Health Record–Linked Platform for Patient-Clinician Partnership
Source: JMIR Mhealth Uhealth. 2020 Nov 26;8(11):e11968. doi: 10.2196/11968 (PMC7728534; doi:10.2196/11968)
Supplement: Multimedia Appendix 1 [file mhealth_v8i11e11968_app1.docx]

**Appendix 1**

**Library of PRO measures for CF**

|  | **Reporting Frequency** | | | | | | | |
| --- | --- | --- | --- | --- | --- | --- | --- | --- |
| General | Daily | Other  *(e.g. M,W,F)* | | | | Weekly | |  |
| Fever |  |  | | | |  | |  |
| Energy Level |  |  | | | |  | |  |
| Exercise |  |  | | | |  | |  |
| Fatigue (PROMIS®)^a^ |  |  | | | |  | |  |
| School Missed |  |  | | | |  | |  |
| General Well Being |  |  | | | |  | |  |
| Pulmonary |  | | | | | | |  |
| Shortness of Breath at Rest (Frequency) |  |  | | | |  | |  |
| Shortness of Breath at Rest (Severity) |  |  | | | |  | |  |
| Shortness of Breath with Activity (Frequency) |  |  | | | |  | |  |
| Shortness of Breath with Activity (Severity) |  |  | | | |  | |  |
| Cough Severity |  |  | | | |  | |  |
| Cough Frequency |  |  | | | |  | |  |
| Cough (daytime)- asked in pm |  |  | | | |  | |  |
| Cough (nighttime) – asked in am |  |  | | | |  | |  |
| Sputum Production |  |  | | | |  | |  |
| Chest Pain (Frequency) |  |  | | | |  | |  |
| Chest Pain (Severity) |  |  | | | |  | |  |
| GI and Nutrition |  | | |  | | | |  |
| Abdominal Pain |  |  | | | |  | |  |
| Appetite |  |  | | | |  | |  |
| Enzyme Supplements |  |  | | | |  | |  |
| Calories |  |  | | | |  | |  |
| Weight |  |  | | | |  | |  |
| Stool Frequency |  |  | | | |  | |  |
| Stool Consistency |  |  | | | |  | |  |
| Other Symptoms |  | | | |  | |  |  |
| Nasal Congestion |  | |  | | |  | |  |
| Sinus Pressure |  | |  | | |  | |  |
| Self-Management & Monitoring |  | | | |  | |  |  |
| Home Spirometery |  | |  | | |  | |  |
| Airway Clearance |  | |  | | |  | |  |

^a^Patient-Reported Outcomes Measurement Information System

|  | **Reporting Frequency** | | | | |
| --- | --- | --- | --- | --- | --- |
| General | Daily | | Other  *(e.g. M,W,F)* | | Weekly |
| Fever |  | |  | |  |
| Energy Level |  | |  | |  |
| Sleep – asked in am |  | |  | |  |
| Fatigue (PROMIS®)^a^ |  | |  | |  |
| School Missed |  | |  | |  |
| General Well Being |  | |  | |  |
| Activity Limitation |  | |  | |  |
| Exercise |  | |  | |  |
| Pain |  | | | | |
| Pain Interference (PROMIS®)^a^ |  | |  | |  |
| Abdominal Pain (Severity) |  | |  | |  |
| Abdominal Pain (Frequency) |  | |  | |  |
| Abdominal Pain (Interference) |  | |  | |  |
| Episodic Abdominal Pain |  | |  | |  |
| Joint Pain & Stiffness |  | |  | |  |
| Pain With Stooling |  | |  | |  |
| Anal Pain |  | |  | |  |
| Stool |  | | | | |
| Nocturnal Stools |  | | |  |  |
| Stool Urgency |  | | |  |  |
| Bloody Stools |  | | |  |  |
| Fecal Incontinence |  | | |  |  |
| Stool Frequency |  | | |  |  |
| Stool Consistency |  | | |  |  |
| Surgical Issues |  |  | | | |
| Ostomy Output |  | | |  |  |
| Ostomy Consistency |  | | |  |  |
| Fistula Drainage |  | | |  |  |
| Nutrition |  |  | | | |
| Appetite |  | | |  |  |
| Food Intake |  | | |  |  |
| Hunger Level |  | | |  |  |
| Weight |  | | |  |  |
| Milk Servings |  | | |  |  |
| Sugary Food Servings |  | | |  |  |
| Other Symptoms |  | | |  |  |
| Nausea |  | | |  |  |
| Mouth Sores |  | | |  |  |
| Bloating (Frequency) |  | | |  |  |
| Bloating (Severity) |  | | |  |  |

**Library of PRO Measures for IBD**

^a^Patient-Reported Outcomes Measurement Information System
