# Supplementary material for: Technology-Enabled Health Care Collaboration in Pediatric Chronic Illness: Pre-Post Interventional Study for Feasibility, Acceptability, and Clinical Impact of an Electronic Health Record–Linked Platform for Patient-Clinician Partnership
Source: JMIR Mhealth Uhealth. 2020 Nov 26;8(11):e11968. doi: 10.2196/11968 (PMC7728534; doi:10.2196/11968)
Supplement: Multimedia Appendix 2 [file mhealth_v8i11e11968_app2.docx]

**Appendix 2**

**Parent Visit Engagement Survey**

Please think about your experiences with your healthcare team **today** and mark the box that best describes how the visit went for you. There are no right or wrong answers. Your answers are private; no one on your healthcare team will see them.

|  | 1=Strongly Disagree | 2=Disagree | 3=Disagree a Little | 4=Agree a Little | 5=Agree | 6=Strongly Agree |
| --- | --- | --- | --- | --- | --- | --- |
| 1. I contributed information that was useful to our healthcare team for managing my child’s health. | **☐**  **1** | **☐**  **2** | **☐**  **3** | **☐**  **4** | **☐**  **5** | **☐**  **6** |
| 1. I used data that we tracked between visits to inform the conversation with our healthcare team.* | **☐**  **1** | **☐**  **2** | **☐**  **3** | **☐**  **4** | **☐**  **5** | **☐**  **6** |
| 1. I had as much responsibility as our healthcare team for setting the agenda during the visit. | **☐**  **1** | **☐**  **2** | **☐**  **3** | **☐**  **4** | **☐**  **5** | **☐**  **6** |
| 1. Before the visit, I thought about specific questions to ask our healthcare team about my child’s lab work, growth or health status. | **☐**  **1** | **☐**  **2** | **☐**  **3** | **☐**  **4** | **☐**  **5** | **☐**  **6** |
| 1. Before the visit, I set specific goals for what I wanted to accomplish during our appointment | **☐**  **1** | **☐**  **2** | **☐**  **3** | **☐**  **4** | **☐**  **5** | **☐**  **6** |
| 1. I shared my goals for what I wanted to accomplish at the visit with our healthcare team. | **☐**  **1** | **☐**  **2** | **☐**  **3** | **☐**  **4** | **☐**  **5** | **☐**  **6** |
| 1. Going into this visit, I felt well-informed and on top of my child’s health status. | **☐**  **1** | **☐**  **2** | **☐**  **3** | **☐**  **4** | **☐**  **5** | **☐**  **6** |
| 1. I used the time with our healthcare team to address the issues that matter most to me and my child. | **☐**  **1** | **☐**  **2** | **☐**  **3** | **☐**  **4** | **☐**  **5** | **☐**  **6** |

*Item not included in analysis as it was not assessed at baseline (relates only to 3- and 6-month follow up)

**Parent Care Impact Survey**

Please think about the Orchestra app and mark the box that best describes your experience.

|  | 1=Strongly Disagree | 2=Disagree | 3=Disagree a Little | 4=Agree a Little | 5=Agree | 6=Strongly Agree |
| --- | --- | --- | --- | --- | --- | --- |
| 1. Using Orchestra helped my child’s care team take better care of my child.^a^ | **☐**  **1** | **☐**  **2** | **☐**  **3** | **☐**  **4** | **☐**  **5** | **☐**  **6** |
| 1. Using Orchestra helped me take better care of my child.^a^ | **☐**  **1** | **☐**  **2** | **☐**  **3** | **☐**  **4** | **☐**  **5** | **☐**  **6** |
| 1. Using Orchestra improved the quality of care my child received from our care team.^a^ | **☐**  **1** | **☐**  **2** | **☐**  **3** | **☐**  **4** | **☐**  **5** | **☐**  **6** |
| 1. Orchestra is an important tool in providing my child the best care possible.^a^ | **☐**  **1** | **☐**  **2** | **☐**  **3** | **☐**  **4** | **☐**  **5** | **☐**  **6** |
| Using Orchestra helped my child’s provider and I have more collaborative discussions.^b^ | **☐**  **1** | **☐**  **2** | **☐**  **3** | **☐**  **4** | **☐**  **5** | **☐**  **6** |
| Using Orchestra helped my child’s provider and I work together to make better care decisions.^b^ | **☐**  **1** | **☐**  **2** | **☐**  **3** | **☐**  **4** | **☐**  **5** | **☐**  **6** |
| Using Orchestra helped me to better contribute to setting the agenda for the visit.^c^ | **☐**  **1** | **☐**  **2** | **☐**  **3** | **☐**  **4** | **☐**  **5** | **☐**  **6** |
| Using Orchestra helped me to better contribute to discussions with my child’s provider.^c^ | **☐**  **1** | **☐**  **2** | **☐**  **3** | **☐**  **4** | **☐**  **5** | **☐**  **6** |
| Using Orchestra helped to focus the visit more on me and my child’s needs and questions.^c^ | **☐**  **1** | **☐**  **2** | **☐**  **3** | **☐**  **4** | **☐**  **5** | **☐**  **6** |
| Using Orchestra gave me new ideas to bring up with our provider.^c^ | **☐**  **1** | **☐**  **2** | **☐**  **3** | **☐**  **4** | **☐**  **5** | **☐**  **6** |
| Using Orchestra helped me make suggestions about my child’s treatment plan.^c^ | **☐**  **1** | **☐**  **2** | **☐**  **3** | **☐**  **4** | **☐**  **5** | **☐**  **6** |
| 1. Using Orchestra helped me be better prepared for my child’s office visit.^c^ | **☐**  **1** | **☐**  **2** | **☐**  **3** | **☐**  **4** | **☐**  **5** | **☐**  **6** |
|  |  |  |  |  |  |  |
|  | **1=Strongly Disagree** | **2=Disagree** | **3=Disagree a Little** | **4=Agree a Little** | **5=Agree** | **6=Strongly Agree** |
| 1. Using Orchestra helped me make new observations about my child’s health.^d^ | **☐**  **1** | **☐**  **2** | **☐**  **3** | **☐**  **4** | **☐**  **5** | **☐**  **6** |
| 1. Using Orchestra helped me learn about what makes my child’s disease better or worse.^d^ | **☐**  **1** | **☐**  **2** | **☐**  **3** | **☐**  **4** | **☐**  **5** | **☐**  **6** |
| 1. Using Orchestra gave me ideas about what I can do to improve the symptoms most important to me and my child.^d^ | **☐**  **1** | **☐**  **2** | **☐**  **3** | **☐**  **4** | **☐**  **5** | **☐**  **6** |
| Using Orchestra helped me make more informed decisions about my child’s treatment plan.^e^ | **☐**  **1** | **☐**  **2** | **☐**  **3** | **☐**  **4** | **☐**  **5** | **☐**  **6** |
| Using Orchestra helped my child’s provider make more informed decisions about my child’s treatment plan.^e^ | **☐**  **1** | **☐**  **2** | **☐**  **3** | **☐**  **4** | **☐**  **5** | **☐**  **6** |
| Using Orchestra helped personalize my child’s treatment plan.^e^ | **☐**  **1** | **☐**  **2** | **☐**  **3** | **☐**  **4** | **☐**  **5** | **☐**  **6** |
| Overall, I feel Orchestra is a useful tool for my child’s care.^f^ | **☐**  **1** | **☐**  **2** | **☐**  **3** | **☐**  **4** | **☐**  **5** | **☐**  **6** |
| I want to keep using Orchestra for my child’s care.^f^ | **☐**  **1** | **☐**  **2** | **☐**  **3** | **☐**  **4** | **☐**  **5** | **☐**  **6** |

^a^Care Quality, ^b^Patient-Clinician Collaboration, ^c^Visit Preparation, ^d^Disease Insight, ^e^Treatment Plan Quality, ^f^Perceived Usefulness
